# Characteristics of Joint Use Agreements in School Districts in the United States: Findings From the School Health Policies and Practices Study, 2012

**DOI:** 10.5888/pcd12.140560

**Published:** 2015-04-16

**Authors:** Sherry Everett Jones, Arthur M. Wendel

**Affiliations:** Author Affiliation: Arthur M. Wendel, Centers for Disease Control and Prevention, Atlanta, Georgia.

## Abstract

**Introduction:**

Joint use or shared use of public school facilities provides community access to facilities for varied purposes. We examined a nationally representative sample of school districts in the United States to identify characteristics associated with having a formal joint use agreement (JUA) and with the kinds of uses to which JUAs apply.

**Methods:**

We analyzed data from the 2012 School Health Policies and Practices Study. The response rate for the module containing questions about formal JUAs was 60.1% (N = 630). We used multivariate logistic regression models to examine the adjusted odds of having a formal JUA and χ^2^ analyses to examine differences in district characteristics associated with the uses of the JUA.

**Results:**

Among the 61.6% of school districts with a formal JUA, more than 80% had an agreement for the use of indoor and outdoor recreation facilities; other uses also were identified. JUAs were more common in urban than rural areas, in large than small school districts, and in the West compared with the Midwest, South, and Northeast.

**Conclusion:**

In many districts, school facilities appear to be an untapped resource for community members. Formal JUAs provide an opportunity for shared use while addressing issues of liability, cost, and logistics.

## Introduction

Joint use or shared use of public school facilities provides community access to facilities for varied purposes. Joint use can result from an informal arrangement (eg, unlocking school playgrounds) or a formal agreement or contract, such as between 2 government entities or a government entity and a private party (1,2). Opening school facilities for physical activity is a tool to address obesity and chronic disease (1,3–5); however, school facilities also can provide space for other uses, for example, continuing education, child care services, or health care services (1). Joint use agreements (JUAs) can include the use of a public or private facility located near a school (1,6–8), such as a public park, private health club, performing arts center, library, or health clinic.

Community use of schools takes advantage of existing infrastructure (1,3–7,9) and can promote support for education and educational facilities among community members without school-age children (approximately 55% of households) because they see evidence that tax revenues benefit them (4,7,10). Common barriers to establishing a formal JUA include insufficient partnerships between school districts and potential collaborating parties (6,7), inadequate institutional capacity to support coordination of joint use (7,11), exaggerated concerns about liability (12,13), and costs associated with increased use of facilities (1,3,6,7,14).

A growing literature explores the benefits of JUAs and provides advice on how to implement formal agreements, which could be specific to one facility or encompass an entire school district’s or community’s facilities. Few studies have examined the characteristics of school districts or schools with a JUA or the types of facilities or purposes for the JUA (6,14). This analysis examined school district characteristics associated with having a formal JUA and with the kinds of uses to which JUAs applied.

## Methods

The 2012 School Health Policies and Practices Study (SHPPS) was conducted by the Centers for Disease Control and Prevention (CDC) from October 2011 through August 2012. SHPPS 2012 data address aspects from all elements of the Whole School, Whole Community, Whole Child model: health education; physical education and physical activity; nutrition environment and services; health services; counseling, psychological, and social services; social and emotional climate; physical environment; employee wellness; family engagement; and community involvement (15). This report examined data from the SHPPS district-level Healthy and Safe School Environment questionnaire. A detailed description of the SHPPS 2012 methods has been published (16).

### Sample and survey administration

SHPPS 2006 questionnaires were reviewed to determine content for 2012. CDC performed cognitive testing using telephone interviews for new questions and questions that were modified substantially from SHPPS 2006. Then, draft questionnaires were evaluated by reviewers from federal agencies, national associations, foundations, universities, and businesses nationwide.

During 2010 and 2011, when the SHPPS 2012 sampling frame was constructed, there were 13,588 US public school districts (17). A nationally representative sample of public school districts (N = 1,057) was invited to participate. Eligible districts were those in operation during the time of recruitment and included regional supervisory unions in places where local school boards only provided funding and limited curriculum guidance. Nine districts were deemed ineligible (4 had merged with another sampled district, and 5 did not have their own student body), leaving 1,048 districts in the sample. Sampled districts were asked to identify respondents who were responsible for or most knowledgeable about the component covered in a questionnaire. Most (85.4%) of the district-level questionnaires were completed via web-based self-administration; the remaining 14.6% were completed using self-administered paper and pencil questionnaires. The Healthy and Safe School Environment questionnaire was composed of 4 modules that grouped related items, allowing for different respondents for each module, as appropriate. The response rate for the module containing questions about formal JUAs was 60.1% (n = 630). Most often, respondents for this module self-identified as a superintendent or assistant superintendent (approximately one-third), but titles across districts varied widely (eg, principal, student services, health and safety, and maintenance and grounds). SHPPS 2012 was reviewed by the institutional review boards at both CDC and ICF Macro, Inc, an ICF International Company (contractor engaged for SHPPS 2012) and determined to be exempt.

### District policies and characteristics

This analysis examined 1) whether the district had a formal agreement between the school district and another public or private entity for shared use of school or community property and 2) the facilities to which the joint use agreement applied (“kinds of uses”). SHPPS data were linked with extant data from the Market Data Retrieval (MDR) database (now MCH Strategic Data). The MDR database is updated annually by contacting the districts directly by telephone, primarily, and contains information about individual US school districts. The MDR variables included in this analysis were percentage of students receiving free or reduced-price lunch, percentage of white students, metropolitan status, number of students, and geographic region.

The percentage of students receiving free or reduced-price lunch (range, 0%–99%) and the percentage of white students (range, 0%–100%) were collapsed into 3 categories: 0%–32%, 33%–65%, and 66%–100%. Metropolitan status was defined by the US Census Bureau and describes the size of the community in which the school district resides. Initially there were 8 categories of metropolitan status defined by the US Census Bureau, but because sample sizes in some categories were small, metropolitan status was collapsed into the following 5 categories: central city (large central city and midsized central city), urban fringe of central city, urban fringe of midsized city or large town, small town, and rural (both inside and outside of a metropolitan statistical area). The number of students in the district (range, 43–1,150,000) was collapsed into 5 categories: ≤299, 300–999, 1,000–2,499, 2,500–4,999, and ≥5,000. The state in which the district resided was used to classify districts into regions based on the Census regions: West, Midwest, South, and Northeast.

### Analysis

All analyses were conducted among districts with a response to the question about the existence of a formal JUA (N = 616). Data were weighted to produce national estimates, and analyses were conducted using SUDAAN statistical software (RTI International) to account for weighted data and the complex sampling design. We used χ^2^ analyses to identify district characteristics associated with having a formal JUA. Logistic regression models, controlling for district characteristics found to be significantly associated with JUA in the χ^2^ analyses, examined the adjusted odds of a district having a formal JUA. An examination of the variance inflation factor found that multicollinearity was not present among district characteristics.

This analysis also examined the kinds of uses to which JUAs applied. The subset sample size (districts with a JUA) was too small to accommodate multiple variables in multivariate logistic regression models. Instead, when a χ^2^ analysis found a significant association between district characteristics and the uses to which the JUA applied, pairwise comparisons were conducted using *t* tests, and significant pairwise comparisons were reported. A *P* value less than .05 was considered significant.

## Results

School districts were distributed across all demographic categories included in this analysis (Table 1). Overall, 61.6% of districts had a formal JUA, the prevalence of which varied by metropolitan status, number of students in the district, and region (Table 2).

**Table 1:** District Characteristics,^a^ School Health Policies and Practices Study, 2012

District Characteristic	Unweighted No. (%)^b^
**Percentage of students receiving free or reduced-price lunch**	
0–32	200 (34.6)
33–65	296 (49.6)
66–100	102 (15.8)
**Percentage of white students**	
0–32	80 (10.5)
33–65	108 (17.3)
66–100	417 (72.2)
**Metropolitan status**	
Central city	70 (8.1)
Urban fringe of central city	114 (18.4)
Urban fringe of midsized city or large town	71 (11.5)
Small town	91 (14.2)
Rural	260 (47.8)
**Number of students in the district**	
≤299	77 (14.5)
300–999	161 (27.5)
1,000–2,499	151 (25.3)
2,500–4,999	112 (18.4)
≥5,000	110 (14.3)
**Region**	
West	96 (14.9)
Midwest	223 (37.0)
South	182 (29.7)
Northeast	115 (18.5)

a Among districts with a response to the question about formal joint use agreements (N = 616). Values for n do not add up to 616 when district characteristic information is missing.

b Weighted population estimate.

**Table 2:** Percentage of Districts With a Formal Joint Use Agreement,^a^ School Health Policies and Practices Study, 2012

District Characteristic	% (95% CI)	χ^2^ (*P* Value)	Adjusted Odds Ratio^b^ (95% CI)
**Total**	61.6 (57.5–65.5)	—	—
**Percentage of students receiving free or reduced-price lunch**			
0–32	65.1 (58.6–71.1)	1.0 (.35)	—
33–65	59.0 (52.9–64.8)		
66–100	59.5 (48.4–69.7)		
**Percentage of white students**			
0–32	68.2 (56.0–78.3)	1.0 (.38)	—
33–65	57.0 (46.9–66.4)		
66–100	61.7 (56.8–66.4)		
**Metropolitan status**			
Central city	66.0 (52.0–77.7)	7.3 (<.001)	0.7 (0.3–1.4)
Urban fringe of central city	78.0 (70.2–84.2)		2.0 (1.1–3.6)
Urban fringe of midsized city or large town	73.3 (61.4–82.5)		1.7 (0.9–3.3)
Small town	52.9 (41.9–63.6)		0.9 (0.5–1.6)
Rural	53.9 (47.7–60.0)		1 [Reference]
**Number of students in the district**			
≤299	49.9 (38.8–61.1)	8.6 (<.001)	1 [Reference]
300–999	60.2 (52.6–67.5)		1.8 (1.0–3.1)
1,000–2,499	52.1 (44.1–59.9)		1.3 (0.7–2.4)
2,500–4,999	71.6 (62.9–79.0)		3.0 (1.5–6.0)
≥5,000	81.2 (71.7–88.1)		6.5 (2.8–14.9)
**Region**			
West	73.3 (64.4–80.6)	3.5 (.02)	1 [Reference]
Midwest	61.3 (54.2–67.9)		0.6 (0.3–1.0)
South	55.2 (47.6–62.5)		0.3 (0.2–0.6)
Northeast	63.0 (52.9–72.0)		0.4 (0.2–0.8)

Abbreviation: — , not calculated; CI, confidence interval.

a A formal joint use agreement is an agreement, such as a memorandum of agreement or understanding, between the school district and another public or private entity to jointly use or share either school facilities or community facilities to share costs and responsibilities.

b The multivariate logistic regression model includes metropolitan status, number of students in the district, and region.

The adjusted odds of districts having a JUA was higher among urban fringe of central city districts compared with rural districts (adjusted odds ratio [AOR] = 2.0; 95% confidence interval [CI], 1.1–3.6); compared with districts with 299 or fewer students, the adjusted odds was higher among districts with 300 to 999 students (AOR = 1.8; 95% CI, 1.0–3.1), 2,500 to 4,999 students (AOR = 3.0; 95% CI, 1.5–6.0) and 5,000 or more students (AOR = 6.5; 95% CI, 2.8–14.9). Compared with districts in the West, the adjusted odds of having a JUA were lower among districts in the Midwest (AOR = 0.6; 95% CI, 0.3–1.0), South (AOR = 0.3; 95% CI, 0.2–0.6), and Northeast (AOR = 0.4; 95% CI, 0.2–0.8).

### Indoor/outdoor recreation facilities and library services

Among districts with a JUA, most often the JUA applied to indoor (82.1%) and outdoor (84.3%) recreation facilities (Table 3). Having a JUA for indoor recreation facilities varied by the percentage of students receiving free or reduced-price lunch (*P* = .02), metropolitan status (*P* = .04), and region (*P* = .005). The percentage of districts with a JUA for indoor recreation facilities was higher among districts with 0% to 32% of students who received free or reduced-price lunch (88.0%) than among districts in which 66% to 100% received free or reduced-price lunch (69.9%); higher in urban fringe of central city districts (91.1%) than in rural districts (75.9%); and higher among districts in the Northeast (93.7%) than among districts in the West (80.1%), Midwest (79.8%), and South (78.7%) (Table 3).

**Table 3:** Among Districts With a Formal Joint Use Agreement,^a^ Prevalence of the Uses Related to Indoor Recreation Facilities, Outdoor Recreation Facilities, and Library Services, School Health Policies and Practices Study, 2012

District Characteristics	Indoor Recreation Facilities		Outdoor Recreation Facilities		Library Services	
	% (95% CI)	χ^2^ (*P* Value)	% (95% CI)	χ^2^ (*P* Value)	% (95% CI)	χ^2^ (*P* Value)
**Total**	82.1 (77.7–85.8)	—	84.3 (80.3–87.5)	—	23.9 (19.7–28.8)	—
**Percentage of students receiving free or reduced-price lunch**						
0–32	88.0 (81.1–92.6)	3.9 (.02)	87.7 (80.9–92.3)	1.5 (.24)	16.2 (10.8–23.5)	3.0 (.049)
33–65	80.4 (73.3–86.0)		82.8 (76.5–87.8)		28.1 (21.5–35.8)	
66–100	69.9 (57.3–80.1)		78.1 (65.4–87.1)		23.4 (14.3–36.0)	
**Percentage of white students**						
0–32	81.9 (69.3–90.1)	0.0 (.99)	84.9 (72.8–92.2)	0.6 (.53)	26.5 (16.0–40.6)	0.3 (.75)
33–65	81.1 (69.6–89.0)		88.5 (77.9–94.4)		25.9 (16.4–38.4)	
66–100	82.1 (76.3–86.7)		83.2 (78.0–87.3)		22.4 (17.6–28.1)	
**Metropolitan status**						
Central city	84.5 (69.9–92.8)	2.5 (.04)	77.2 (62.4–87.3)	7.0 (<.001)	22.2 (11.8–37.7)	1.3 (.25)
Urban fringe of central city	91.1 (83.7–95.3)		92.4 (83.8–96.9)		18.4 (11.7–27.7)	
Urban fringe of midsized city or large town	85.7 (74.3–92.6)		98.0 (87.2–99.7)		16.1 (7.7–30.7)	
Small town	78.8 (64.3–88.5)		79.8 (66.2–88.8)		29.1 (17.7–44.9)	
Rural	75.9 (67.3–82.8)		77.0 (69.8–83.0)		28.1 (21.4–35.8)	
**Number of students in the district**						
≤299	58.7 (39.0–76.0)	1.9 (.12)	60.9 (46.0–74.1)	3.5 (.008)	44.2 (30.3–59.0)	1.9 (.11)
300–999	82.9 (74.1–89.2)		83.9 (75.1–90.0)		22.9 (15.0–33.4)	
1,000–2,499	86.3 (76.4–92.5)		84.6 (75.2–90.9)		19.4 (12.2–29.5)	
2,500–4,999	86.4 (76.7–92.5)		91.7 (83.5–96.0)		17.7 (10.7–27.8)	
≥5,000	85.4 (75.3–91.9)		89.2 (79.6–94.6)		24.3 (15.7–35.7)	
**Region**						
West	80.1 (68.0–88.4)	4.4 (.005)	84.0 (74.4–90.5)	1.1 (.37)	28.5 (18.7–40.8)	0.4 (.78)
Midwest	79.8 (71.3–86.3)		80.8 (73.3–86.6)		22.6 (15.8–31.2)	
South	78.7 (69.3–85.8)		84.6 (76.3–90.4)		24.6 (16.9–34.5)	
Northeast	93.7 (86.1–97.3)		90.0 (79.9–95.3)		21.1 (13.4–31.6)	

Abbreviation: CI, confidence interval.

a Among the 61.6% (n = 385) of districts with a formal joint use agreement. A formal joint use agreement is an agreement, such as a memorandum of agreement or understanding, between the school district and another public or private entity to jointly use or share either school facilities or community facilities to share costs and responsibilities.

Having a JUA for outdoor recreation facilities varied by metropolitan status (*P* < .001) and by the number of students in the district (*P* = .008). The percentage of districts with a JUA for outdoor recreation facilities was higher among urban fringe of midsized city or large town districts (98.0%) than among central city (77.2%), small town (79.8%), and rural (77.0%) districts, and it was higher among urban fringe of central city districts (92.4%) than among central city (77.2%) and rural (77.0%) districts. The percentage of districts with this kind of JUA was lower among districts with 299 or fewer students (60.9%) than among districts with 300 to 999 (83.9%); 1,000 to 2,499 (84.6%); 2,500 to 4,999 (91.7%); and 5,000 or more (89.2%) students (Table 3).

Having a JUA for library services varied by the percentage of students receiving free or reduced-price lunch (*P* = .049). The percentage of districts with a JUA for library services was higher among districts in which 33% to 65% of students received free or reduced-price lunch (28.1%) than among districts in which 0% to 32% of students received free or reduced-price lunch (16.2%) (Table 3).

### Care for school-aged children, adult education, and health care services

Having a JUA for before- or after-school care for school-aged children varied by the number of students in the district (*P* = .004) and by the percentage of white students (*P* = .03). The percentage of districts with this kind of JUA was lower among districts with 299 or fewer students (47.1%) than among districts with 1,000 to 2,499 (70.2%); 2,500 to 4,999 (74.9%); and 5,000 or more (78.4%) students; it was lower among districts with 300 to 999 students (57.8%) than among districts with 2,500 to 4,999 students (74.9%) and 5,000 or more students (78.4%). The percentage of districts with this kind of JUA was higher among districts with 0% to 32% of students who were white (78.2%) than among districts with 66% to 100% of students who were white (63.4%).

Having a JUA for adult education varied by the number of students in the district (*P* = .005). The percentage of districts with this kind of JUA was higher among districts with 5,000 or more (68.6%) students than among districts with 299 or fewer (38.4%); 300 to 999 (41.6%); 1,000 to 2,499 (48.4%); and 2,500 to 4,999 students (50.6%).

Having a JUA for health care services varied by the percentage of students receiving free or reduced-price lunch (*P* = .005), the percentage of white students (*P* = .005), the number of students in the district (*P* = .001), and region (*P* < .001). The percentage of districts with a JUA for health care services was lower among districts with 0% to 32% of students who received free or reduced-price lunch (12.8%) than among districts in which 66% to 100% received free or reduced-price lunch (35.4%). The percentage of districts with this kind of JUA was higher among districts with 0% to 32% of students who were white (31.4%) than among districts with 66% to 100% of students who were white (16.2%). The percentage of districts with this kind of JUA was lower among districts with 300 to 999 students (8.8%) than among districts with 299 or fewer (25.5%); 1,000 to 2,499 (22.8%); 2,500 to 4,999 (19.9%); and 5,000 or more (34.8%) students, and the percentage was lower among districts with 2,500 to 4,999 students (19.9%) than among districts with 5,000 or more students (34.8%). The percentage of districts with a JUA for health care services was higher in the South (34.5%) than in the Northeast (9.7%) and Midwest (15.4%) and higher in the West (23.7%) than in the Northeast (9.7%) (Table 4).

**Table 4:** Among Districts With a Formal Joint Use Agreement,^a^ Prevalence of the Uses Related to Preschool/Infant Childcare, Before- or After-School Care for School-Aged Children, Adult Education, and Health Care Services, School Health Policies and Practices Study, 2012

District Characteristics	Preschool/Infant Childcare		Before- or After-School Care for School-Aged Children		Adult Education		Health Care Services	
	% (95% CI)	χ^2^ (*P* Value)	% (95% CI)	χ^2^ (*P* Value)	% (95% CI)	χ^2^ (*P* Value)	% (95% CI)	χ^2^ (*P* Value)
**Total**	41.1 (36.0–46.5)	—	67.0 (61.7–72.0)	—	49.3 (44.1–54.6)	—	21.0 (17.1–25.4)	—
**Percentage of students receiving free or reduced-price lunch**								
0–32	36.9 (28.3–46.3)	2.3 (.10)	71.8 (63.4–78.9)	1.7 (.19)	44.2 (35.3–53.4)	1.3 (.27)	12.8 (7.7–20.5)	5.3 (.005)
33–65	47.2 (39.3–55.2)		62.2 (54.4–69.5)		53.7 (46.7–60.5)		21.4 (15.5–28.7)	
66–100	32.6 (21.7–45.7)		71.0 (56.3–82.2)		49.2 (34.9–63.7)		35.4 (24.3–48.3)	
**Percentage of white students**								
0-32	44.6 (30.6–59.6)	0.4 (.68)	78.2 (65.3–87.3)	3.6 (.03)	62.8 (47.9–75.5)	2.1 (.13)	31.4 (20.2–45.3)	5.3 (.005)
33–65	36.6 (26.0–48.7)		74.2 (60.9–84.1)		51.6 (38.4–64.6)		34.4 (24.1–46.5)	
66–100	41.6 (35.1–48.4)		63.4 (57.0–69.4)		46.8 (40.7–53.1)		16.2 (11.9–21.7)	
**Metropolitan status**								
Central city	44.4 (29.9–59.9)	1.3 (.28)	77.2 (60.6–88.1)	2.2 (.07)	56.3 (41.2–70.3)	0.3 (.87)	34.0 (20.1–51.3)	1.9 (.11)
Urban fringe of central city	40.4 (30.1–51.5)		74.8 (65.3–82.5)		47.7 (35.9–59.7)		16.2 (9.8–25.5)	
Urban fringe of mid-sized city or large town	26.6 (15.6–41.5)		70.5 (55.9–81.8)		50.3 (36.0–64.6)		12.2 (5.5–25.0)	
Small town	38.0 (25.2–52.8)		62.3 (47.9–74.7)		49.6 (35.9–63.3)		26.3 (16.0–40.1)	
Rural	44.6 (36.7–52.9)		58.9 (49.3–67.8)		46.7 (38.2–55.4)		21.5 (15.8–28.7)	
**Number of students in the district**								
≤299	39.6 (25.0–56.4)	1.9 (.11)	47.1 (28.2–66.8)	4.0 (.004)	38.4 (21.0–59.5)	3.7 (.005)	25.5 (14.2–41.4)	4.7 (.001)
300–999	35.0 (26.2–45.0)		57.8 (47.7–67.2)		41.6 (31.7–52.3)		8.8 (4.5–16.6)	
1,000–2,499	43.5 (32.0–55.6)		70.2 (59.1–79.3)		48.4 (37.4–59.5)		22.8 (14.3–34.1)	
2,500–4,999	35.2 (25.3–46.5)		74.9 (64.1–83.2)		50.6 (40.3–60.8)		19.9 (12.2–30.8)	
≥5,000	53.8 (42.2–65.0)		78.4 (67.3–86.4)		68.6 (57.2–78.2)		34.8 (24.9–46.3)	
**Region**								
West	36.6 (25.5–49.4)	0.9 (.45)	65.8 (51.7–77.5)	1.2 (.31)	48.1 (35.5–60.8)	0.4 (.74)	23.7 (15.4–34.8)	5.7 (<.001)
Midwest	37.6 (30.0–45.9)		62.2 (53.0–70.6)		47.9 (39.2–56.6)		15.4 (10.1–22.7)	
South	47.4 (37.1–58.0)		67.3 (57.5–75.9)		54.3 (44.4–63.9)		34.5 (25.6–44.6)	
Northeast	42.6 (30.4–55.7)		75.9 (63.9–84.9)		47.1 (35.1–59.5)		9.7 (4.6–19.1)	

Abbreviation: —, not calculated; CI, confidence interval.

a Among the 61.6% (n = 385) of districts with a formal joint use agreement. A formal joint use agreement is an agreement, such as a memorandum of agreement or understanding, between the school district and another public or private entity to jointly use or share either school facilities or community facilities to share costs and responsibilities.

## Discussion

Overall, 61.6% of school districts nationwide had a formal JUA. These agreements were more common in urban areas, in large school districts, and in the West. Among districts with a JUA, more than 80% had an agreement addressing the use of indoor and outdoor recreation facilities, but other uses included before- or after-school care for school-aged children (67.0%), adult education (49.3%), preschool or infant childcare (41.1%), library services (23.9%), and health care services (21.0%).

That so many districts lacked a JUA suggests the need to eliminate barriers to and promote the benefits of such agreements. Eliminating “silo planning” among districts and other government officials and taking deliberate action toward building relationships and addressing separate bureaucracies, which may not structurally be set up to collaborate, could help (1,6,7,11). Some districts might lack staff experienced in implementing JUAs and be reluctant to attempt them (7,11). The design of some public schools may not easily accommodate community use (1,4,7), and a lack of park and recreation infrastructure (4,6,7) limits options for use of those facilities for many schools. Anticipating joint use when designing new school buildings or renovations and in developing community parks and other infrastructure could facilitate joint use of facilities (1,4,6,7).

State laws requiring that schools be available for community use may increase the prevalence of JUAs (1,4,12). In 2010, eight states required and 37 states and the District of Columbia allowed, but did not require, schools to be available for community use (18). Laws that provide legal protection for districts allowing community use may allay district concerns about liability (1,4,6,12,13). A 50-state survey of liability risks for after-hours use of public school property found that liability risks are “unlikely to be substantial enough to justify denying recreational access” (12) and that fears over liability are exaggerated (12,13).

A well-constructed agreement addresses liability concerns and issues such as responsibilities for maintenance and repairs; insurance, risk management, and liability; staffing and communications; property and facilities being used; and security (1,4,8,14,19). Implementing a JUA may enhance support for education and educational facilities when community members see tangible evidence that community tax revenue benefits households without children in the local public school system (4,7,10).

Costs associated with increased use of school facilities vary. Schools often subsidize joint use, recouping little of the costs (6,20), but costs may be less than anticipated. In a district with more than 143,000 students, a community-sponsored after-school program to increase physical activity successfully improved participation in the program but did not result in significantly higher school operating expenses (3). District budget structure, use of community property (eg, a park) rather than school property, or other factors may allow some districts to more easily absorb costs associated with joint use; however, to reduce concerns about costs, as well as actual financial strain on school systems, JUAs can articulate responsibilities for facility operation, staffing, and maintenance and repair costs. JUAs that clearly articulate these responsibilities may make JUAs more appealing to school and district administrators (1,3,6,14).

Other studies have found that rural, nonwhite, and lower-income communities often lack community recreational facilities (4,21–23). SHPPS found that JUAs are less common in rural districts, suggesting that establishing JUAs, which most often address recreation facilities, might improve these disparities. Increasing the number of JUAs in the South, where only 55.2% of districts had a JUA, might help address disproportionately low “healthy lifestyle characteristics” among populations in the South (24).

Overall, having a JUA did not vary by the percentage of students receiving free or reduced-price lunch or the percentage of white students, but specific use did vary: indoor recreation facilities, library services, and health care services varied by the percentage of students who received free or reduced-price lunch, and before- and after-school care for school-aged children and health care services varied by the percentage of white students. Larger districts were the most likely to have a JUA overall and to have one that addressed before- or after-school care, adult education, and health care services. Whether these types of JUAs result from larger overall budgets, exceptional need, better partner collaboration, or some other factor is not clear. More information about how larger and high-need communities successfully navigate JUAs would be useful.

In communities without a JUA, assistance may be needed on how to initiate and sustain JUAs and how to fund staffing needed to develop and implement a JUA. Resources are available from organizations such as ChangeLab Solutions (http://changelabsolutions.org), Bridging the Gap (www.rwjf.org/en/research-publications/find-rwjf-research/2012/02/joint-use-agreements-creating-opportunities-for-physical-activit.html), Safe Routes to School National Partnership (http://saferoutespartnership.org), Public Health Law Center at William Mitchell College of Law (http://publichealthlawcenter.org), and Center for Cities and Schools (http://citiesandschools.berkeley.edu/joint-use.html).

This study has several limitations. First, these data are based on self-report. Underreporting or overreporting of the existence or content of JUA cannot be determined nor can misclassification of school district characteristics. Second, SHPPS did not analyze district JUAs or examine barriers or incentives to implementing JUAs. Third, SHPPS inquired about JUAs for school or community use. Determining the percentage of agreements for shared use of school property versus other community property was not possible. Fourth, among districts with a JUA, SHPPS did not inquire about the proportion of schools in those districts to which a JUA agreement applied, nor did SHPPS examine the extent to which community members use school facilities as a result of the JUA. Finally, formal JUAs are likely an underestimate of the extent to which schools are available for community use, because districts without a JUA may still allow for community use of school facilities.

This study found that 61.6% of school districts nationwide had a formal JUA. For many communities, school facilities may be an untapped resource that could offer opportunities for physical activity, child care, adult education, library services, or health care where they may be lacking — in small districts, districts in rural areas, and districts in the South, Midwest, and Northeast. For communities needing assistance on how to initiate and sustain JUAs, a growing literature on JUAs could help address common barriers such as concerns about liability, costs, and logistics to establishing JUAs.
